# 5-Ethyl-4a-meth­oxy-1,3-dimethyl-4a,5-dihydro­benzo[*g*]pteridine-2,4(1*H*,3*H*)dione

**DOI:** 10.1107/S1600536809020856

**Published:** 2009-06-10

**Authors:** Petra Ménová, Václav Eigner, Radek Cibulka, Jan Čejka, Hana Dvořáková

**Affiliations:** a Department of Organic Chemistry, Institute of Chemical Technology, Prague, Technická 5, 166 28, Prague 6, Czech Republic; b Department of Solid State Chemistry, Institute of Chemical Technology, Prague, Technická 5, 166 28, Prague 6, Czech Republic; c Central Laboratories, Institute of Chemical Technology, Prague, Technická 5, 166 28, Prague 6, Czech Republic

## Abstract

The title compound, C_15_H_18_N_4_O_3_, was formed by the reaction of methanol with 5-ethyl-1,3-dimethyl­alloxazinium perchlorate. Its structure mimics those of possible flavin inter­mediates in flavoenzymes. The heterocyclic rings are substituted with methyl, ethyl and meth­oxy groups. The central tricyclic skeleton is bent due to the presence of an *sp* ^3^ C atom. There are weak inter­molecular C—H⋯O inter­actions in the structure, forming a three-dimensional network.

## Related literature

in the context of this article, a C4a-adduct is a compound with a nucleophile covalently bound to atom C4a of the flavin fragment; isoalloxazines are natural flavin derivatives, alloxazines are their isomers. For the biological relevance of C4a-adducts in flavoenzymes, see: Palfey & Massey (1998 ▶); Massey (2000 ▶); Müller (1991 ▶). For the preparation of C4a-isoalloxazine adducts, see: Kemal & Bruice (1976 ▶); Kemal *et al.* (1977 ▶); Hoegy & Mariano (1997 ▶). For the crystal structures of isoalloxazine adducts, see: Bolognesi *et al.* (1978 ▶). For the crystal structures of reduced isoalloxazines, see: Werner & Rönnquist (1970 ▶); Norrestam & Von Glehn (1972 ▶). For puckering parameters, see: Cremer & Pople (1975 ▶). For the extinction correction, see: Larson (1970 ▶).
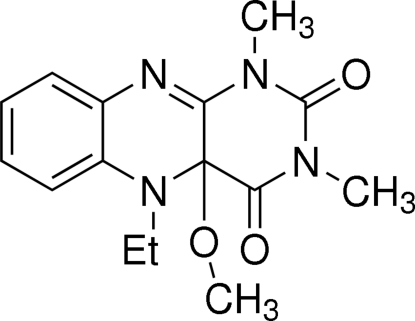


## Experimental

### 

#### Crystal data

C_15_H_18_N_4_O_3_
*M* *_r_* = 302.33
Monoclinic, 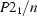

*a* = 10.3958 (2) Å
*b* = 12.7174 (2) Å
*c* = 10.9421 (2) Å
β = 100.4727 (16)°
*V* = 1422.53 (4) Å^3^
*Z* = 4
Cu *K*α radiation
μ = 0.83 mm^−1^
*T* = 150 K
0.50 × 0.28 × 0.15 mm

#### Data collection

Oxford Diffraction Xcalibur diffractometer
Absorption correction: analytical (de Meulenaer & Tompa, 1965 ▶) *T*_min_ = 0.76, *T*_max_ = 0.88
18511 measured reflections
2996 independent reflections
2692 reflections with *I* > 2σ(*I*)
*R*_int_ = 0.025

#### Refinement

*R*[*F* ^2^ > 2σ(*F* ^2^)] = 0.041
*wR*(*F* ^2^) = 0.121
*S* = 0.99
2996 reflections
200 parameters
H-atom parameters constrained
Δρ_max_ = 0.23 e Å^−3^
Δρ_min_ = −0.21 e Å^−3^

### 

Data collection: *CrysAlis CCD* (Oxford Diffraction, 2005 ▶); cell refinement: *CrysAlis RED* (Oxford Diffraction, 2005 ▶); data reduction: *CrysAlis RED*; program(s) used to solve structure: *Superflip* (Palatinus & Chapuis, 2006 ▶); program(s) used to refine structure: *CRYSTALS* (Betteridge *et al.*, 2003 ▶); molecular graphics: *ORTEP-3* (Farrugia, 1997 ▶); software used to prepare material for publication: *CRYSTALS* and *PARST97* (Nardelli, 1997).

## Supplementary Material

Crystal structure: contains datablocks global, I. DOI: 10.1107/S1600536809020856/fb2153sup1.cif

Structure factors: contains datablocks I. DOI: 10.1107/S1600536809020856/fb2153Isup2.hkl

Additional supplementary materials:  crystallographic information; 3D view; checkCIF report

## Figures and Tables

**Table 1:** Hydrogen-bond geometry (Å, °)

*D*—H⋯*A*	*D*—H	H⋯*A*	*D*⋯*A*	*D*—H⋯*A*
C4—H42⋯O1^i^	0.96	2.43	3.3230 (18)	155
C14—H141⋯O21^ii^	0.94	2.56	3.3999 (18)	149
C19—H191⋯O6^iii^	0.97	2.46	3.3021 (18)	146

Symmetry codes: (i) 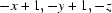; (ii) 
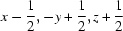
; (iii) 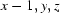.

**Fig. 1.**
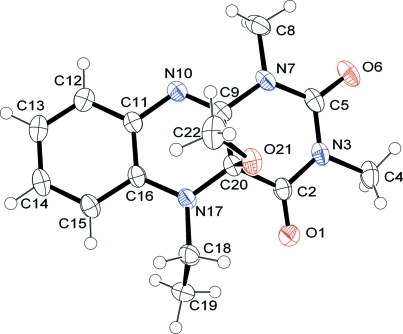
The title molecule with the displacement ellipsoids drawn at the 50% probability level. The H atoms are shown as spheres of arbitrary radius.

**Fig. 2.**
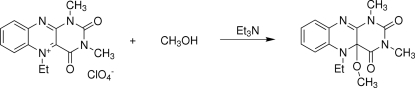
Formation of the adduct by the reaction of 5-ethyl-1,3-dimethylalloxazinium perchlorate with methanol.

## supplementary crystallographic information

### Comment

Flavinium salts (both, isoalloxazinium and alloxazinium) represent suitable models (Müller, 1991; Kemal & Bruice, 1976; Kemal *et al.*, 1977) of natural flavin derivatives which are important cofactors in many types of oxido-reductases and monooxygenases (Massey, 2000; Palfey & Massey, 1998). Similarly to natural flavins, flavinium salts react easily with various nucleophiles (water, methanol, primary amines *etc*.) with the formation of the covalent C4a-adducts (C4a-adduct means a compound with the covalently bound nucleophile to the C4a-atom of the flavin fragment; see Kemal & Bruice, 1976; Kemal *et al.*, 1977; Hoegy & Mariano, 1997). The C4a-adducts of flavins are important intermediates of the reactions catalyzed by flavoenzymes.

In this paper, the first crystal structure of the C4a-adduct of alloxazinium salt (Figs. 1 and 2) is reported. The adduct is formed by the reaction of methanol with 5-ethyl-1,3-dimethylalloxazinium perchlorate (Fig. 2). By this reaction, the hybridization of C20 atom (C4a atom in IUPAC numbering of alloxazine moiety) is changed from *sp*^2^ to *sp*^3^ (Fig. 2). This change of hybridization causes a folding of the tricyclic alloxazine skeleton. The value of the interplanar angle between the plane determined by the C2, N3, C5, and N7 atoms and the plane determined by the C9, N10, C11, C12, C13, C14, C15, C16, and N17 atoms is 15.69 (5)°. This angle is larger in comparison with that found in the case of the similar adducts of C-nucleophiles with isoalloxazine derivatives; *e.g.* the angle between the analogous planes in 4a,5-dihydro-4a-isopropyl-3,10-dimethylisoalloxazine (Bolognesi *et al.*, 1978) is only 6.85 (9)°. The observed 'butterfly' arrangement of the tricyclic alloxazine subunit in the title compound corresponds to the structure of dihydroflavins already published by Werner & Rönnquist (1970) and Norrestam & Von Glehn (1972).

Due to the *sp*^3^ hybridization, C20 atom is shifted out of the alloxazine plane by 0.313 (1)Å. On the other hand, the values of the bond angles around C20 are different from those expected for an *sp*^3^ carbon atom, probably due to the rigidity of the dihydroalloxazine system. The conformation of the ring 1 (C2, N3, C5, N7, C9, C20) is between ^5^H_6_ and E_6_. The conformation of the ring 2 (C9, N10, C11, C16, N17, C20) is between ^5^S_6_ and E_6_, rather closer to E_6_. The distances, angles and puckering parameters (Cremer & Pople, 1975) were calculated using *PARST97* (Nardelli, 1999).

Three weak intermolecular C—H···O interactions were found forming a three-dimensional network.

### Experimental

The crystals of the title compound were obtained from a solution of 1,3-dimethyl-5-ethylalloxazinium perchlorate (20 mg, 0.054 mmol) and dry triethylamine (7.5 µl, 0.054 mmol) in dry methanol (1.8 ml). Single crystals suitable for analysis were grown overnight directly from the reaction mixture. M. p. 384 - 386 K.

### Refinement

The H atoms were found in the Δρ map and initially refined with the restraints on the bond lengths and angles to regularize their geometry (C_methyl_—H  = 0.96 (2), C_methylene_—H = 0.97 (2), C_aryl_ = 0.93 (2) Å. *U*_iso_(H) = 1.5 U_eq_C_methyl_ or 1.2 U_eq_C_methylene/aryl_. After the convergement the geometrical restraints were substituted by the geometrical constraints.

^1^H NMR (pyridine-d5; 600 MHz): 1.57 (t, 3H; CH_2_C*H*_3_), 2.82 (s, 3H; OCH_3_), 3.31 (s, 3H; 3 N–CH_3_), 3.56 (s, 3H; 1 N–CH_3_), 3.58–3.62 (m, 1H; 5 N–C*H*_2_CH_3_), 4.17–4.21 (m, 1H; 5 N–C*H*_2_CH_3_), 7.03–7.07 (m, 2H; 6,8–CH), 7.33 (t, ^2^J = 7.20 Hz, 1H; 7–CH), 7.63 (d, ^2^J = 7.14 Hz, 1H; 9–CH). ^13^C NMR (pyridine-d5; 150 MHz): 50.9 (OCH_3_), 82.2 (4a–C).

### Crystal data

**Table:**

C_15_H_18_N_4_O_3_	*F*(000) = 640
*M**_r_* = 302.33	*D*_x_ = 1.412 Mg m^−^^3^
Monoclinic, *P*2_1_/*n*	Melting point = 384–386 K
Hall symbol: -P 2yn	Cu *K*α radiation, λ = 1.54184 Å
*a* = 10.3958 (2) Å	Cell parameters from 11727 reflections
*b* = 12.7174 (2) Å	θ = 4–77°
*c* = 10.9421 (2) Å	µ = 0.83 mm^−^^1^
β = 100.4727 (16)°	*T* = 150 K
*V* = 1422.53 (4) Å^3^	Prism, colourless
*Z* = 4	0.50 × 0.28 × 0.15 mm

### Data collection

**Table:**

Oxford Diffraction Xcalibur diffractometer	2996 independent reflections
graphite	2692 reflections with *I* > 2σ(*I*)
Detector resolution: 8.1917 pixels mm^-1^	*R*_int_ = 0.025
φ and ω scans	θ_max_ = 77.5°, θ_min_ = 5.4°
Absorption correction: analytical (de Meulenaer & Tompa, 1965)	*h* = −13→13
*T*_min_ = 0.76, *T*_max_ = 0.88	*k* = −15→15
18511 measured reflections	*l* = −12→13

### Refinement

**Table:**

Refinement on *F*^2^	Secondary atom site location: difference Fourier map
Least-squares matrix: full	Hydrogen site location: difference Fourier map
*R*[*F*^2^ > 2σ(*F*^2^)] = 0.041	H-atom parameters constrained
*wR*(*F*^2^) = 0.121	Modified Sheldrick (2008) *w* = 1/[σ^2^(*F*^2^) + (0.08*P*)^2^ + 0.33*P*], where *P* = [max(*F*_o_^2^,0) + 2*F*_c_^2^]/3
*S* = 0.99	(Δ/σ)_max_ = 0.0003
2996 reflections	Δρ_max_ = 0.23 e Å^−^^3^
200 parameters	Δρ_min_ = −0.21 e Å^−^^3^
0 restraints	Extinction correction: Larson (1970), Equation 22
Primary atom site location: structure-invariant direct methods	Extinction coefficient: 29 (5)

### Fractional atomic coordinates and isotropic or equivalent isotropic displacement parameters (Å^2^)

**Table:**

	*x*	*y*	*z*	*U*_iso_*/*U*_eq_	
O1	0.43813 (9)	0.38054 (7)	0.10771 (8)	0.0341	
C2	0.53277 (11)	0.36250 (9)	0.18746 (11)	0.0269	
N3	0.65728 (10)	0.37227 (8)	0.16350 (9)	0.0295	
C4	0.67024 (14)	0.39751 (11)	0.03524 (12)	0.0394	
C5	0.77221 (12)	0.37049 (9)	0.25223 (12)	0.0308	
O6	0.87775 (9)	0.38486 (8)	0.22244 (10)	0.0413	
N7	0.75942 (9)	0.35479 (8)	0.37412 (10)	0.0293	
C8	0.87888 (12)	0.36402 (11)	0.46793 (13)	0.0388	
C9	0.63877 (10)	0.35461 (8)	0.41330 (11)	0.0249	
N10	0.63770 (9)	0.37433 (8)	0.52755 (9)	0.0272	
C11	0.51698 (11)	0.37383 (9)	0.56668 (11)	0.0260	
C12	0.51588 (13)	0.38898 (10)	0.69269 (11)	0.0317	
C13	0.39963 (14)	0.39033 (10)	0.73696 (11)	0.0340	
C14	0.28241 (13)	0.37940 (9)	0.65335 (12)	0.0333	
C15	0.28184 (12)	0.36561 (9)	0.52737 (12)	0.0301	
C16	0.39920 (11)	0.36095 (8)	0.48170 (10)	0.0248	
N17	0.40282 (9)	0.34548 (8)	0.35602 (9)	0.0257	
C18	0.27877 (11)	0.31653 (11)	0.27437 (11)	0.0328	
C19	0.19478 (12)	0.41186 (13)	0.22860 (13)	0.0412	
C20	0.52333 (10)	0.31848 (9)	0.31740 (10)	0.0249	
O21	0.53556 (8)	0.20753 (6)	0.29281 (7)	0.0295	
C22	0.54526 (16)	0.14088 (10)	0.39936 (13)	0.0417	
H41	0.7567	0.3805	0.0256	0.0569*	
H42	0.6530	0.4708	0.0177	0.0574*	
H43	0.6097	0.3549	−0.0197	0.0574*	
H81	0.8696	0.3196	0.5369	0.0560*	
H82	0.8932	0.4348	0.4948	0.0553*	
H83	0.9525	0.3400	0.4345	0.0558*	
H121	0.5991	0.3989	0.7479	0.0377*	
H131	0.3999	0.3991	0.8214	0.0392*	
H141	0.2022	0.3814	0.6805	0.0402*	
H151	0.2003	0.3581	0.4738	0.0353*	
H181	0.2289	0.2703	0.3207	0.0369*	
H182	0.2982	0.2774	0.2036	0.0371*	
H191	0.1071	0.3889	0.1944	0.0565*	
H192	0.1917	0.4597	0.2970	0.0566*	
H193	0.2316	0.4495	0.1641	0.0564*	
H221	0.5453	0.0697	0.3721	0.0593*	
H222	0.6286	0.1545	0.4591	0.0602*	
H223	0.4725	0.1509	0.4432	0.0599*	

### Atomic displacement parameters (Å^2^)

**Table:**

	*U*^11^	*U*^22^	*U*^33^	*U*^12^	*U*^13^	*U*^23^
O1	0.0330 (5)	0.0392 (5)	0.0302 (4)	0.0028 (4)	0.0063 (3)	0.0028 (3)
C2	0.0302 (6)	0.0222 (5)	0.0303 (5)	0.0018 (4)	0.0110 (4)	−0.0008 (4)
N3	0.0308 (5)	0.0291 (5)	0.0322 (5)	0.0028 (4)	0.0156 (4)	0.0040 (4)
C4	0.0483 (7)	0.0396 (7)	0.0363 (7)	0.0079 (6)	0.0236 (6)	0.0081 (5)
C5	0.0302 (6)	0.0237 (6)	0.0425 (7)	0.0025 (4)	0.0174 (5)	0.0041 (4)
O6	0.0300 (5)	0.0432 (5)	0.0566 (6)	0.0001 (4)	0.0231 (4)	0.0068 (4)
N7	0.0218 (5)	0.0302 (5)	0.0379 (5)	0.0017 (4)	0.0107 (4)	0.0035 (4)
C8	0.0229 (6)	0.0440 (7)	0.0491 (7)	0.0016 (5)	0.0056 (5)	0.0067 (6)
C9	0.0226 (5)	0.0207 (5)	0.0328 (6)	0.0009 (4)	0.0085 (4)	0.0025 (4)
N10	0.0256 (5)	0.0260 (5)	0.0306 (5)	0.0007 (3)	0.0068 (4)	0.0020 (4)
C11	0.0271 (6)	0.0218 (5)	0.0305 (6)	−0.0003 (4)	0.0093 (4)	0.0007 (4)
C12	0.0393 (6)	0.0270 (6)	0.0295 (6)	0.0004 (5)	0.0082 (5)	−0.0004 (4)
C13	0.0481 (7)	0.0271 (6)	0.0307 (6)	0.0007 (5)	0.0179 (5)	0.0006 (4)
C14	0.0388 (6)	0.0261 (6)	0.0407 (6)	−0.0025 (5)	0.0227 (5)	−0.0008 (5)
C15	0.0285 (6)	0.0265 (6)	0.0381 (6)	−0.0040 (4)	0.0137 (5)	−0.0029 (4)
C16	0.0272 (5)	0.0202 (5)	0.0292 (5)	−0.0020 (4)	0.0111 (4)	−0.0005 (4)
N17	0.0220 (4)	0.0279 (5)	0.0286 (5)	−0.0020 (4)	0.0082 (3)	−0.0034 (4)
C18	0.0247 (5)	0.0412 (7)	0.0334 (6)	−0.0084 (5)	0.0074 (4)	−0.0088 (5)
C19	0.0238 (5)	0.0612 (9)	0.0373 (6)	0.0020 (5)	0.0020 (5)	−0.0044 (6)
C20	0.0244 (5)	0.0228 (5)	0.0293 (5)	−0.0003 (4)	0.0097 (4)	−0.0006 (4)
O21	0.0350 (4)	0.0223 (4)	0.0342 (4)	0.0002 (3)	0.0141 (3)	−0.0013 (3)
C22	0.0614 (9)	0.0254 (6)	0.0435 (7)	0.0023 (6)	0.0231 (6)	0.0045 (5)

### Geometric parameters (Å, °)

**Table:**

O1—C2	1.2124 (15)	C12—H121	0.969
C2—N3	1.3723 (15)	C13—C14	1.3914 (19)
C2—C20	1.5476 (15)	C13—H131	0.930
N3—C4	1.4696 (15)	C14—C15	1.3886 (18)
N3—C5	1.3961 (17)	C14—H141	0.935
C4—H41	0.949	C15—C16	1.4012 (16)
C4—H42	0.962	C15—H151	0.944
C4—H43	0.955	C16—N17	1.3965 (14)
C5—O6	1.2138 (15)	N17—C18	1.4758 (14)
C5—N7	1.3786 (16)	N17—C20	1.4347 (14)
N7—C8	1.4650 (16)	C18—C19	1.524 (2)
N7—C9	1.3973 (14)	C18—H181	0.983
C8—H81	0.961	C18—H182	0.972
C8—H82	0.950	C19—H191	0.966
C8—H83	0.956	C19—H192	0.969
C9—N10	1.2771 (16)	C19—H193	0.985
C9—C20	1.5149 (15)	C20—O21	1.4464 (13)
N10—C11	1.3977 (15)	O21—C22	1.4300 (15)
C11—C12	1.3944 (16)	C22—H221	0.953
C11—C16	1.4060 (16)	C22—H222	1.002
C12—C13	1.3813 (18)	C22—H223	0.975
			
O1—C2—N3	121.04 (11)	C13—C14—C15	120.70 (11)
O1—C2—C20	123.43 (10)	C13—C14—H141	120.9
N3—C2—C20	115.33 (10)	C15—C14—H141	118.4
C2—N3—C4	117.11 (11)	C14—C15—C16	120.83 (12)
C2—N3—C5	125.69 (10)	C14—C15—H151	118.1
C4—N3—C5	116.89 (10)	C16—C15—H151	121.1
N3—C4—H41	108.0	C11—C16—C15	117.99 (10)
N3—C4—H42	110.9	C11—C16—N17	119.44 (10)
H41—C4—H42	110.2	C15—C16—N17	122.56 (10)
N3—C4—H43	108.2	C16—N17—C18	117.02 (9)
H41—C4—H43	109.2	C16—N17—C20	120.34 (9)
H42—C4—H43	110.3	C18—N17—C20	118.40 (9)
N3—C5—O6	120.78 (12)	N17—C18—C19	112.69 (10)
N3—C5—N7	117.02 (10)	N17—C18—H181	108.8
O6—C5—N7	122.17 (12)	C19—C18—H181	108.7
C5—N7—C8	116.60 (10)	N17—C18—H182	109.0
C5—N7—C9	123.15 (10)	C19—C18—H182	109.6
C8—N7—C9	118.62 (10)	H181—C18—H182	108.1
N7—C8—H81	108.0	C18—C19—H191	109.3
N7—C8—H82	111.0	C18—C19—H192	110.0
H81—C8—H82	110.2	H191—C19—H192	109.2
N7—C8—H83	110.0	C18—C19—H193	110.3
H81—C8—H83	108.3	H191—C19—H193	109.3
H82—C8—H83	109.3	H192—C19—H193	108.6
N7—C9—N10	117.91 (10)	C2—C20—C9	110.62 (9)
N7—C9—C20	115.48 (10)	C2—C20—N17	112.87 (9)
N10—C9—C20	126.22 (10)	C9—C20—N17	110.34 (9)
C9—N10—C11	117.84 (10)	C2—C20—O21	99.18 (8)
N10—C11—C12	118.13 (11)	C9—C20—O21	109.84 (9)
N10—C11—C16	121.37 (10)	N17—C20—O21	113.53 (9)
C12—C11—C16	120.49 (11)	C20—O21—C22	114.97 (9)
C11—C12—C13	120.89 (12)	O21—C22—H221	108.1
C11—C12—H121	117.9	O21—C22—H222	110.8
C13—C12—H121	121.2	H221—C22—H222	108.6
C12—C13—C14	119.06 (11)	O21—C22—H223	112.2
C12—C13—H131	120.3	H221—C22—H223	108.8
C14—C13—H131	120.6	H222—C22—H223	108.2

### Hydrogen-bond geometry (Å, °)

**Table:**

*D*—H···*A*	*D*—H	H···*A*	*D*···*A*	*D*—H···*A*
C4—H42···O1^i^	0.96	2.43	3.3230 (18)	155
C14—H141···O21^ii^	0.94	2.56	3.3999 (18)	149
C19—H191···O6^iii^	0.97	2.46	3.3021 (18)	146

Symmetry codes: (i) −*x*+1, −*y*+1, −*z*; (ii) *x*−1/2, −*y*+1/2, *z*+1/2; (iii) *x*−1, *y*, *z*.
